# Large Scale Genome Analysis Shows that the Epitopes for Broadly Cross-Reactive Antibodies Are Predominant in the Pandemic 2009 Influenza Virus A H1N1 Strain

**DOI:** 10.3390/v5112796

**Published:** 2013-11-19

**Authors:** Edgar E. Lara-Ramírez, Aldo Segura-Cabrera, Ma Isabel Salazar, Mario A. Rodríguez-Pérez, Xianwu Guo

**Affiliations:** 1 Laboratory of Molecular Biomedicine, Genomics and Biotechnology Center, National Polytechnic Institute, Col. Narciso Mendoza, Reynosa 88710, Mexico; E-Mails: aldosegura@gmail.com (A.S.-C.); drmarodriguez@hotmail.com (M.A.R.-P.); gxianwu@yahoo.com (X.G.); 2 Laboratory for Cellular Immunology and Immunopathogenesis, Department of Immunology, National School for Biological Sciences (ENCB), National Polytechnic Institute, Mexico City 11340, Mexico; E-mail: isalazarsan@yahoo.com

**Keywords:** influenza A virus, H1N1, pandemic, epitopes, genome comparison

## Abstract

The past pandemic strain H1N1 (A (H1N1)pdm09) has now become a common component of current seasonal influenza viruses. It has changed the pre-existing immunity of the human population to succeeding infections. In the present study, a total of 14,210 distinct sequences downloaded from National Center for Biotechnology Information (NCBI) database were used for the analysis. The epitope compositions in A (H1N1)pdm09, classic seasonal strains, swine strains as well as highly virulent avian strain H5N1, identified with the aid of the Immune Epitope DataBase (IEDB), were compared at genomic level. The result showed that A (H1N1) pdm09 contains the 90% of B-cell epitopes for broadly cross-reactive antibodies (EBCA), which is in consonance with the recent reports on the experimental identification of new epitopes or antibodies for this virus and the binding tests with influenza virus protein HA of different subtypes. Our analysis supports that high proportional EBCA depends on the epitope pattern of A (H1N1)pdm09 virus. This study may be helpful for better understanding of A (H1N1)pdm09 and the production of new influenza vaccines.

## 1. Introduction

A novel H1N1 virus variant, A (H1N1)pdm09, initiated in Mexico and USA in April 2009 and caused the first 21st century pandemic influenza. Currently, this virus, as a regular seasonal influenza virus, is co-circulating in many parts of the world along with classic seasonal influenza viruses, so that the current seasonal virus composition (classic seasonal viruses and new pandemic virus) has apparently changed due to the pandemic outbreak. The data from the Centers for Disease Control and Prevention (CDC) showed that the pandemic strains accounted for 26.5% and 4.3% of the “influenza A” infection cases in USA respectively in the 2010–2011 and 2011–2012 influenza seasons [1]. This trend leads to a possible change of pre-existing immunity in human population to subsequent infections. Therefore, the comparison of B-cell epitope composition of A (H1N1)pdm09 with other human influenza strains at genomic level could provide valuable reference data for the control of new influenza infection. 

## 2. Results and Discussion

The epitope composition comparisons among A (H1N1)pdm09, recent classic seasonal influenza strain, swine strains and “bird flu” virus (H5N1 in human) were performed, as shown in Table 1. All detected epitopes (21) in the novel pandemic strain were conserved in the swine viral group due to its swine origin. The 12 epitopes were shared by all the viral tested groups. The 85% (18/21) of epitopes from A (H1N1)pdm09 were shared with H5N1, consistent with the previous experiments on the antibodies produced from the patients infected by A (H1N1)pdm09 or the people inoculated with A (H1N1)pdm09 vaccines [2,3]. The comparison of the amino acid sequences of NA from different strains also showed that pandemic strains have more similarity of NA to H5N1 strains than to classic swine strains and human seasonal strains. They even have a slightly higher identity with H5N1 strains than with the H1N1 strains circulating in 1918 and in 1934, indicating that more epitopes on NA could be shared between the A (H1N1)pdm09 and H5N1 strains. Indeed, the A (H1N1)pdm09 also shared five epitopes on HA protein with H5N1 strains, although they belong to different serotypes (H1 and H5). These five epitopes were also shared with the strains seasonal H1N1, swine H1N1. Two epitopes on HA in the pandemic strains were shared with the strains seasonal H1N1, seasonal H3N2 and swine H1N1. It can thus be inferred that a majority of epitopes (90%) (19/21) in A (H1N1)pdm09 are the epitopes for inducing broadly cross-reactive antibodies (EBCA) (Table 1). If the epitopes with nested sequences in the database were considered to be the same epitopes, a similar result was still obtained in which 88.9% (16/18) of the epitopes in A (H1N1)pdm09 are EBCA (Table S1). The 82% (9/11) of epitopes on NA, HA and M2 of A (H1N1)pdm09 could induce the broadly cross-reactive neutralizing antibodies. Due to the presence of highly proportional EBCA epitopes, it is understandable why A (H1N1)pdm09 produced cross-reactive antibodies that can bind H1, H5 and H3 influenza viruses [2,4]. This feature in A (H1N1)pdm09 is distinctive from the classic seasonal strains. Therefore, a question arises: is this feature a typical inherent trait for A (H1N1)pdm09 or just a consequence of distinct immune response from humans to new strains?

**Table 1:** B-cell epitopes of A (H1N1)pdm09 shared with other influenza strains and subtypes.

Strains					HA	NA	M2	M1	NS1	NS2	NP	PA	PB1	PB1-F2	PB2	Total
pH1N1 *	sH1N1	sH3N2	SIV	H5N1	2		1	2			4		2		1	12
pH1N1	sH1N1	sH3N2	SIV		2		1	2			5		2		1	13
pH1N1	sH1N1		SIV	H5N1	5	2	1	2			4		2		1	17
pH1N1	sH1N1		SIV		6	2	1	2			5		2		1	19
pH1N1			SIV	H5N1	5	3	1	2			4		2		1	18
pH1N1			SIV		7 (1 dis)	3	1	2			5		2		1	21

*: pH1N1 indicates novel pandemic H1N1 strain. The similar signals are: sH3N2, human seasonal H3N2 strains; sH1N1, human seasonal H1N1 strains; SIV, swine influenza A strains; H5N1, human highly virulent “bird flu” strains; dis, discontinuous epitope.

Our genomic analysis support the proposal that this feature is an attribute of A (H1N1)pdm09. This means that the high proportional EBCAs principally depend on the epitope pattern (epitope composition and immunogenic strengthen of epitopes) of virus. To date, the research focuses more on the most important immune protein, HA. More studies on the identification of new epitopes from HA and from other proteins are needed. Such information should be very helpful to provide a conclusive answer to this question. On the other hand, it could be a distinct immune response of humans to a new strain. A model for human immune response induced after infection or vaccination with this pandemic strain has been proposed [4]. When the epitope composition of A (H1N1)pdm09 in comparison with that from recent seasonal influenza A viruses was dramatically altered and a considerable proportion of dominant and variable epitopes recognized by the majority of preexisting influenza-specific memory B cells were missed, there is a greater probability to activate the rare memory B cells that recognize conserved HA epitopes because of less competition for this recognition. This hypothesis needs to be confirmed with other antigenic shift strains. 

EBCAs have the following characteristics. First, they are sited at the conserved region. These epitopes in different strains need to keep similar conformation that could be recognized by the same antibody. Therefore, they are usually located at the sites with strong functional constrains, as a consequence of purifying selection processes that limit diversity. Second, these epitopes usually produce low antibody titers. It can be inferred that if they have strong immunogenicity, the different groups of strains might fuse into the same serotype. Third, the immune response to such epitopes depends on the individuals, indicating that the components or conditions for immunogenic response differ among individuals [5,6,7,8].

Most epitopes in A (H1N1)pdm09 were located at the stem region of HA. The epitopes on the stem and proximal-membrane regions of surface proteins are more conserved and prone to induce the broadly cross-reactive antibodies [9,10,11,12]. However, some EBCA epitopes were recently found to be located at the head of HA [4,13]. Meanwhile, experiments on mice confirmed the cross protection by cross-reactive monoclonal antibody or vaccines [10,14,15,16]. All the aforementioned information suggest the possibility to produce a universal vaccine in the future. However, some characteristics of EBCAs still remain a challenge in terms of obtaining the effective prophylaxis and therapy. Currently, the research and application of such epitopes have already produced a promising result [17,18]. 

## 3. Methods

The sequences for test were downloaded from the Influenza Virus Resource in the National Center for Biotechnology Information (NCBI) [19]. The search for epitopes was based on each chromosomal fragment, not on complete genome, in order to include more information. The following parameters for each viral protein of A (H1N1)pdm09 were used.

Virus Species = Influenza A AND (Subtype = H1N1).

Host = Human

Year FROM 2009 TO 2010

Complete sequences only

INCLUDE = Pandemic only

EXCLUDE = Lab strains

EXCLUDE = Flu Project

Collapse similar sequences

In order to search for the sequences in the other groups of strains, it is necessary to change the corresponding parameters. After elimination of identical sequences, a total of 14210 distinct sequences, including the 1148 distinct fragments from A (H1N1)pdm09, were downloaded for the present study. The sequences of recent seasonal influenza strains (sH1N1 and sH3N2) were collected from the strains circulating in the previous 20 years (1988–2008) before the pandemic [20].

The epitopes of influenza strains were extracted from the Immune Epitope DataBase (IEDB) [21]. All epitopes present in IEDB have been confirmed by experiments. All sequences of each viral protein were used to search for epitopes with the help of epitope conservancy analysis tool provided by IEDB. Considering that the structural constraints associated with their immune recognition could be similar across species due to B-cell epitopes generally defined in different species [20], B-cell epitopes identified in the context of any host organism were included. The epitopes with nested sequences were considered as separate epitopes because they could represent different epitopes (some epitopes in IEDB contain more than 40 amino acids). Even though the nested epitopes represent a unique individual epitope, the result of epitope comparison among strains would not be affected because the tests for all viruses were performed under the same condition. The shortest analyzed sequence of epitope was limited to be ≥ 5 residues for linear epitopes and ≥ 4 residues for discontinuous epitopes. After obtaining the required B-cell epitopes in the group of strains at certain duration, the comparisons of epitopes on specific protein among the groups of strains were manually implemented. Only the epitopes with 100% identity were considered. If the same protein of influenza A strains, such as H1, H3 and H5, has the identical amino acid sequence of an epitope, these strains share the epitope. If the same proteins from the same serotype of influenza A strains has the exact amino acids at the exact sites of a discontinuous epitope, the strains share the discontinuous epitope. For example, some discontinuous epitopes on H1 from A (H1N1)pdm09 were shared with some epitopes on H1 from the classic seasonal strains. 

## 4. Conclusions

Our analysis showed that EBCAs are predominant in A (H1N1)pdm09, corresponding to the recent reports on the experiments for identification of new epitopes or antibodies. It seems to be an attribute of A (H1N1)pdm09. This result could provide reference to design strategies for effective prophylaxis and therapy.

## Supplementary Files

Supplementary File 1 Supplemental table S1 (PDF, 27 KB)
