# Supplementary material for: Large Scale Genome Analysis Shows that the Epitopes for Broadly Cross-Reactive Antibodies Are Predominant in the Pandemic 2009 Influenza Virus A H1N1 Strain
Source: Viruses. 2013 Nov 19;5(11):2796–802. doi: 10.3390/v5112796 (PMC3856415; doi:10.3390/v5112796)
Supplement: Supplementary File 1 — Supplemental table S1 (PDF, 27 KB) [file viruses-05-02796-s001.pdf]

Table S1: B-cell epitopes on the pandemic genomes

Proteins	Epitope ID	Epitope Sequences
**HA**	20836	*GLFGAIAGF**
**HA**	20838	*GLFGAIAGF*IE
**HA**	97348	*GLFGAIAGF*IEGGW
**HA**	133590	GMVDGWYG
**HA**	62335	SVSSFERFEIFPK
**HA**	133748	YNAELLV
**NA**	97177	*AELPF*
**NA**	97246	DNSIRIGSKGDVFVIREPFISCSHLECRTFFLTQGALLNDKH
**NA**	97748	VNSDTVGWSWPDG*AELPF*TID
**M2**	97650	SLLTEVET
**M1**	37043	LKTRPILSPLTKGILGFVFTLTVPSERGLQRRRFVQNALNGNGD
**M1**	129610	PLTKGILGFVFT
**NP**	15381	FDERRNKYLEEHPSAGKDPKKTGGPI
**NP**	17539	FQTAAQR
**NP**	45359	NPGNAEIEDLIFLAR
**NP**	58052	SGGNTNQQR
**NP**	67436	TYQRTRALV
**PB1**	97407	KAGLLVSDGGPNLY
**PB1**	97236	DAVATTHSWIPKRNRSIL
**PB2**	97293	EVLTGNLQTLKIRVHEGYEEFTMVGRRATAILR
**HA**	91060	N140, P152, K168, K169

- Italic letters represent the core sequence of nested epitopes.
